# Direct Quantitative Detection and Identification of Lactococcal Bacteriophages from Milk and Whey by Real-Time PCR: Application for the Detection of Lactococcal Bacteriophages in Goat's Raw Milk Whey in France

**DOI:** 10.1155/2011/594369

**Published:** 2011-10-13

**Authors:** Mai Huong Ly-Chatain, Loïc Durand, Véronique Rigobello, Annabelle Vera, Yann Demarigny

**Affiliations:** Department of Food Industry and Quality, ISARA-Lyon, 23 Jean Baldassini, 69364 Lyon Cedex 07, France

## Abstract

The presence of *Lactococcus* bacteriophages in milk can partly or completely inhibit milk fermentation. To prevent the problems associated with the bacteriophages, the real-time PCR was developed in this study for direct detection from whey and milk of three main groups of *Lactococcus* bacteriophages, c2, 936, and P335. The optimization of DNA extraction protocol from complex matrices such as whey and milk was optimized allowed the amplification of PCR without any matrix and nontarget contaminant interference. The real-time PCR program was specific and with the detection limit of 10^2^ PFU/mL. The curve slopes were −3.49, −3.69, and −3.45 with the amplification efficiency estimated at 94%, 94%, and 98% and the correlation coefficient (*R* ^2^) of 0.999, 0.999, and 0.998 for c2, 936 and P335 group, respectively. This method was then used to detect the bacteriophages in whey and goat's raw milk coming from three farms located in the Rhône-Alpes region (France).

## 1. Introduction

Raw milk is a complex microbial ecosystem containing different microorganisms, spoiling bacteria, pathogens, and technological flora such as lactic acid bacteria. The last bacteria participate in the cheese-making process and ripening [1]. Raw milk may also contain the bacteriophages of lactic acid bacteria which could present a risk during dairy processing. The lactic acid bacteria may be infected by the bacteriophages leading to a slower acidification process or the end of it [2]. The disappearance of several flora may modify ripening processes. The contamination of bacteriophages can result from the environment, raw milk, machines, and airborne bacteria. Moreover, some strains of bacteriophages are resistant to pasteurization [3].

The whey separated after the first fermentation can be used for the next fermentation in cheese making, (backslopping). This practice allows the reproduction of a large number of microbial flora technologically [4]. But this practice may reproduce also the contamination of bacteriophages which are suspected to be a cause of acidification incidents, especially in cheese processing using raw milk [1, 3]. It seemed essential to determine the biodiversity of bacteriophages in raw milk and whey to understand the incidents related to acidification.

The diversity of bacteriophages in most important dairy strains (*Streptococcus thermophilus*, *Lactobacillus*, and *Lactococcus*) was studied in several countries: in Canada [5], in Spain [6], in Argentina [7], and in Belarus [8]. In these studies, lactococcal phages were mostly studied for *Lactococcus lactis* application in many processes. Three main lytic lactococcal phage groups were identified: c2, P335, and 936 [5].

The presence of lactococcal bacteriophages in milk and whey has been detected until now by various methods: spot, double layer, and flux cytometry [8–10]. The identification of these bacteriophages can be done by PCR and multiplex PCR. The bacteriophages are first isolated from milk and whey on an agar plate by the double layer method. The phage propagation is then inoculated in M17 and may be concentrated by polyethylene glycerol for PCR analyses or electronic microscopy. 

Real-time PCR is used as a rapid and sensitive technique to detect and identify microorganisms directly. This technique has recently been developed to detect c2 and 936 phage groups from airborne. Real-time PCR has not been yet developed for the use of direct identification and quantification of lactococcal bacteriophages from milk and whey samples. One of the challenges in real-time PCR is the optimization of DNA extraction from complex matrices such as milk and whey. The extraction steps should maximize the viral genome and eliminate the inhibitions for PCR reaction. The choice of primers and PCR conditions is also important. In the present work, we developed a real-time PCR to directly detect and quantify three groups of lactococcal bacteriophages in milk and whey samples using the nonspecific fluorescent dye SYBR Green. The technique is applied to detect bacteriophages in goat's raw milk and whey collected in France. So far, it should be noted that the detection of bacteriophages has not been studied in whey and goat milk in literature.

## 2. Materials and Methods

### 2.1. Bacterial Strains and Bacteriophages

The bacteriophages and bacterial strains used in this study are listed in Table 1. The bacterial strains *Lactococcus lactis *were cultured in M17 broth supplemented with 0.5% lactose at 30°C.

### 2.2. Bacteriophage Multiplication and Titration

The bacteriophages were added in cultures of host bacteria at early stationary phase (OD = 0.7). The cultures infected with phage suspensions were then incubated at 30°C for 5 h. After replication, the phage suspension was inoculated at 4°C overnight before being centrifuged (5000 g for 15 min). The supernatant was decontaminated of the bacteria by filtration through sterile membrane filters (0.22 *μ*m, Millipore). The phage concentration was quantified by the double agar layer plaque assay method [8]. Initial concentration phage stocks about 10^9^ PFU/mL (PFU: plaque forming unit) were stored at 4°C.

### 2.3. Development of Extraction Protocol of Bacteriophages DNA from Milk or Whey Milk

The extraction protocol of bacteriophage DNA was based on the *QIAamp DNA Stool Mini Kit *(Quiagen 51504) with the following modifications: one mL of sample (milk or whey or pure culture) was incubated in 5 mL of buffer ASL (Quiagen) at 70°C for 10 min. The sample was homogenized by vortex for 1 min before centrifugation (5000 g, 5 min). The supernatant incubated with the InhibitEX for 1 min at 25°C was centrifuged at 5000 g for 5 min. The supernatant was mixed with 1 volume of isopropanol (−20°C). The sample was centrifuged at 5000 g for 5 min. The supernatant was then discarded, and the pellet was suspended a second time in 200 *μ*L of buffer ASL and 200 *μ*L of buffer AL (Quiagen) with proteinase K at 70°C for 10 min. Ethanol absolute was added (vol/vol). The sample was continued with step 13th of the protocol for isolation in the *QIAamp DNA Stool Mini Kit*. The DNA was extracted by a double elution using 2 × 50 *μ*L Buffer AE.

### 2.4. Real-Time PCR

#### 2.4.1. Optimization of Primer Concentration

The primers selected according to Cruz Martín et al. [11] (Table 2) were provided from Sigma (France). To determine the optimal concentration of primer in PCR, preliminary tests were performed by combining the primers in three final concentrations: 200, 400, and 800 nM at different hybridization temperatures: 55, 58, and 62°C.

#### 2.4.2. Real-Time PCR Conditions

Amplification and detection were performed with the Minicon system (Bio-Rad, France). The PCR reaction mixture contained 12.5 *μ*L of SYBR Green PCR master mix (Quiagen, France), 9,5 *μ*L of water, 1 *μ*L of each primer (0.4 *μ*M as final concentration), and 1 *μ*L of DNA in a 25 *μ*L final volume. The reaction was done under the following conditions: 98°C for 30 sec and 40 cycles of 95°C for 15 s, 62°C for 30 sec. 

All samples were automatically processed for melting curve analysis of amplified DNA using the software CFX Manager, version 1.5 (Bio-Rad). Standard curves were made by plotting the Ct (cycle threshold) values of the real-time PCR performed on dilution series of DNA. Positive and negative controls were added in each assay. The slope (*s*) of the standard curve was used for determining the PCR efficiency (*E*) in conformity with *E* = 10  (−1/*s*) − 1 [12]. The amplification of DNA was verified by conventional PCR [6].

The PCR products were separated on a 2% agarose in TAE buffer (40 mM Tris-acetate, 1 mM EDTA), stained with ethidium bromide and visualized under UV light.

#### 2.4.3. Real-Time PCR in Pure Culture

A stock of bacteriophages at 10^9^ PFU/mL was diluted at 10^0^ to 10^8^ PFU/mL. The extraction of 1 mL of each concentration was done using the protocol described above, and the real-time PCR protocol was used.

The experiment was repeated in triplicate.

#### 2.4.4. Specific PCR

Extraction and PCR were done with 1 mL of targeted phages at 10^5^ PFU/mL mixed with 10^7^ PFU/mL of other phages. The amplification, the Ct values, and agarose gel were compared between the mixed phages and the stock of one phage at the same concentration.

The assay of real-time PCR was verified with other bacteriophages, P2 and bIL67 of c2 group, sk1 and P270 of P335 group, and P008 and bIL170 of 936 group.

#### 2.4.5. Real-Time PCR in Artificially Contaminated Whey Samples

The presence of bacteriophages from whey collected on a farm was detected by spot method. Whey without bacteriophages was artificially contaminated with one of three bacteriophages c2, P335, and 936 as following. A pure culture of bacteriophages at 10^9^ PFU/mL was diluted at 10^1^ to 10^8^ PFU/mL in M17. 100 *μ*L of each dilution was added in 900 *μ*L of whey to have the whey artificially contaminated with a bacteriophage at different concentrations: 10^0^ to 10^7^ PFU/mL. The extraction of 1 mL of whey artificially contaminated and 1 mL of pure culture at the same concentration of phage was done using the protocol described above. To evaluate the interference of matrix, the Ct values obtained for the detection of different concentrations of bacteriophages in pure culture were compared with values obtained for the detection of the same concentration in the whey. To evaluate the performance of the extraction protocol, the Ct value obtained from the extraction of a tenfold serial dilution of pure culture at 10^8^ PFU/mL was compared with the Ct value obtained from the dilution of a tenfold serial of DNA extracted from a stock of 10^8^ PFU/mL. 

The experiment was repeated in triplicate.

#### 2.4.6. Detection of Lactococcus Bacteriophages in Whey and in Goat's Milk

Real-time PCR was applied to detect the presence of bacteriophages in whey and in goat's raw milk collected on three farms of the Rhône-Alpes region (France). The milk collected from different days was used for fermentation of lactic acid cheese-making. The whey collected after fermentation was mixed with the raw milk for the next fermentation (backslopping).

The milk or whey samples contaminated by the greatest number of bacteriophages were used to isolate bacteriophages. The bacteriophage infection was tested by spot method by using the host bacteria in Table 1 and the *Lactococcus lactis* strains isolated from raw milk collected above (the data of bacterial isolation was not showed in this study for the confidential raison). The phage isolation was done by double-layer according to Quiberoni et al. [7]. The identification of isolated bacteriophages was verified by multiplex-PCR [6].

## 3. Results

### 3.1. Real-Time PCR

The tests were performed on the phage c2 which corresponded to the group predominantly found in cow's milk industries. The similar data obtained for the bacteriophages 936 and P335 were not showed.

#### 3.1.1. Real-Time PCR Conditions

The optimum conditions of PCR were first tested on serial tenfold dilutions of DNA of phage c2 at different hybridization temperatures: 55, 58, and 62°C and by using different concentrations of primers 200, 400, and 800 nM. The results of slope (*s*), efficacy amplification (*E*), and the squared correlation coefficient (*R* ^2^) of each temperature were given in Table 3. A hybridization temperature of 62°C and 400 nM of primers gave the best results of amplification. The squared correlation coefficient was estimated to be 0,997 with the slope close to −3,475 and an amplification efficacy of 94%. The tests were then performed on the DNA of phages 936 and P335. This program was applied to detect the phages in pure culture.

#### 3.1.2. Detection of Bacteriophages in Pure Culture

**Figure 1:**
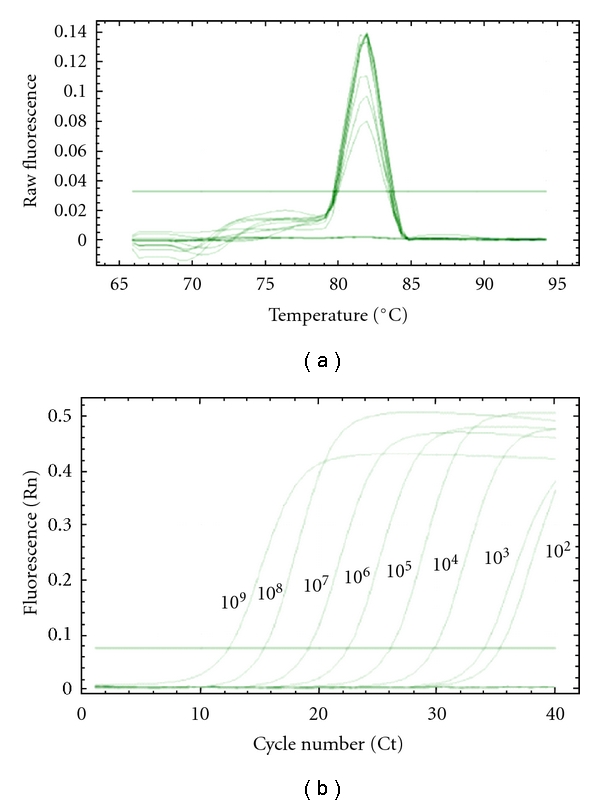
Melting curve analyses of the amplified PCR product of c2 (a) and the curve represents fluorescent changes over cycles of a 10-fold serial dilution of c2 (b).

**Figure 2:**
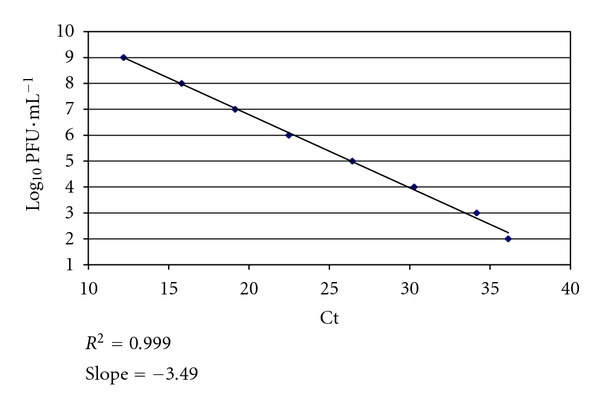
Standard curves for bacteriophage c2 using SYBR in the real-time PCR.

**Figure 3:**
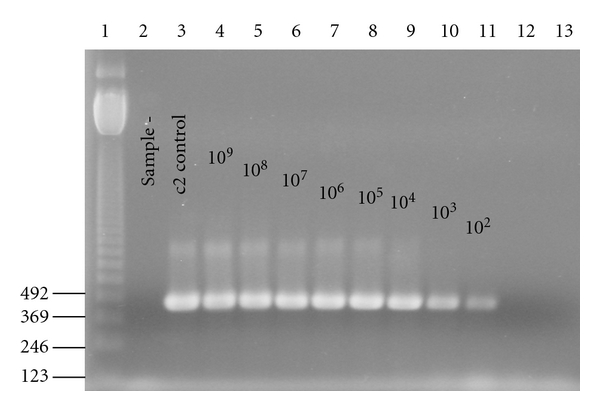
The real-time PCR of c2 at different concentrations on agarose gel. Lane 1: DNA ladder (pb). Lane 2: negative control. Lane 3: positive control (10^9^ UFP/mL of c2 amplified by conventional PCR). Lane 4–13: concentration of c2 from 10^9^ to 10^0^ UFP/mL.

The standard curves of each group bacteriophage were established. A linear relationship was observed between the Ct values and the starting quantity of phages measured with the double agar layer plaque assay method. For each group of phage, the extraction and the real-time PCR were performed with a series of dilutions from 10^8^ to 10^0^ PFU/mL of pure cultures. These results were used for the calibration curves of real time PCR that allowed the bacteriophage quantification. The amplification profiles and melting curves of phage c2 obtained with *Tm *was 81.5°C (Figure 1). The melting curves showed the specificity of primers using the SYBR Green (Figure 1(a)). The amplification was only detected for phage c2. When no bacteriophage was added to the reaction tube, no Ct value was obtained (Figure 1(b)). A linear relationship was observed between the Ct values and the bacteriophage quantity measured with the double agar layer plaque assay method (Figure 2). The quantification was limited at 10^2^ phages/mL. These results were confirmed by gel agarose (Figure 3).

The slopes of the curves were, respectively, −3.49, −3.69, and −3.45 with a correlation coefficient (*R* ^2^) of 0.999 for c2, 936 and 0.998 for P335. PCR amplification efficiencies (*E*) were then estimated to be 93, 93 and 95%, respectively.

#### 3.1.3. Specificity of qPCR

**Figure 4:**
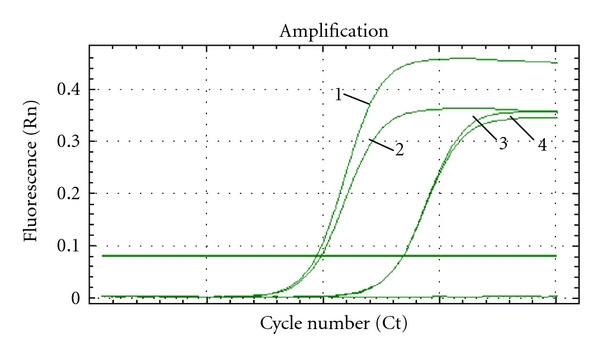
Amplification of DNA bacteriophages in the sample containing c2 alone at 10^5^ UFP/mL (1) and in the sample mixed of c2 at 10^5^ UFP/mL (2) with 10^7^ UFP/mL of 936 (3) and with 10^7^ UFP/mL of P335 (4).

**Figure 5:**
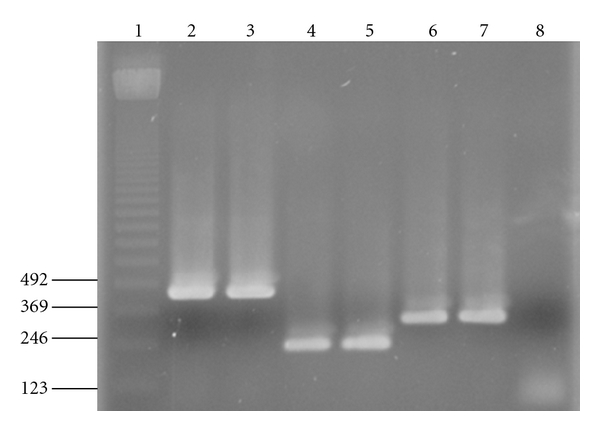
The PCR products of the sample containing the phage alone at 10^5^ UFP/mL and in the sample mixed of three phages. Lane 1: DNA ladder (pb). Lane 2: c2 alone. Lane 3: c2 in the sample mixing three phages. Lane 4: 936 alone. Lane 5: 936 in the sample mixing three phages. Lane 6: P335 alone Lane 7: P335 in the sample mixing three.

The specificity of PCR is the ability of the method to measure exactly a given amount of phages within the sample without interfering with nontargeted contaminants on matrix. The detection (the amplification and the agarose gel result) of a targeted phage from a mixture of the three bacteriophages (c2, 936, and P335) was compared with the detection of this phage in pure culture at the same concentration. For each assay, the quantity 10^5^ PFU/mL of the targeted phage was mixed with other more concentrated phages (10^7^ PFU/mL). An amplification reaction was observed only in reaction tubes containing the expected bacteriophage.  Figure 4 showed the superposition of the amplification curve of c2 alone and c2 in mixing three phages at the same concentration. The presence of other bacteriophages did not influence the amplification reaction. This observation was confirmed by gel electrophoresis (Figure 5). These results confirmed that no interference was observed when the detection of one bacteriophage was performed in presence of the other bacteriophages. Real-time PCR developed above was tested with other bacteriophages P2 and bIL67 of c2 group, sk1 and P270 of P335 group, P008 and bIL170 of 936 group. Similar results were obtained; only the targeted phage was detected in pure culture or in mixing with other phages. The concentration obtained by real-time PCR was identical with that obtained by double layer method (Table 4).

### 3.2. Detection of Bacteriophages in Artificially Contaminated Whey

**Figure 6:**
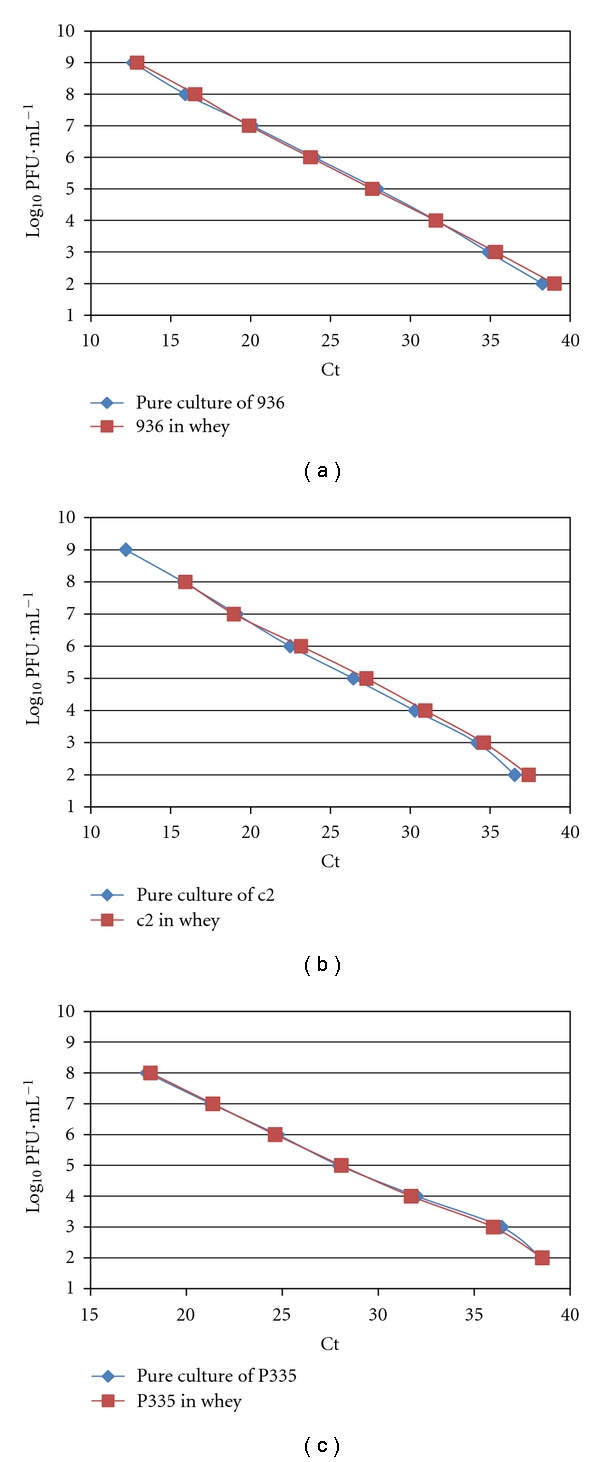
Comparison of standard curves of pure culture and whey artificially contaminated with bacteriophages c2 (a), 936 (b), and P335 (c). Analysis of medium Ct value obtained in relation to bacteriophages concentrations log (PFU/mL).

To evaluate the efficiency of extraction, a pure culture at 10^8^ PFU/mL was diluted tenfold (from 10^8^ PFU/mL to 10^0^ PFU/mL). The Ct value obtained from the extraction of each phage concentration was compared with the Ct obtained from the dilution of tenfold DNA extracted from a pure culture at 10^8^ PFU/mL. The identical curve was obtained (data not showed). To evaluate the effect of matrix on the extraction protocol and PCR reaction, the Ct values obtained from the extraction of artificially contaminated whey and of pure culture at the same concentration of phage were compared. The identical curves obtained with the artificially contaminated whey and pure culture showed no interference of matrix on the PCR amplification (Figure 6). These curves may be used for calibration. For three serial dilutions of pure culture and three serial wheys contaminated with different concentrations of each phage, the detection limit was estimated at 10^2^ PFU/mL for all bacteriophage groups. The presence of lactic acid bacteria in whey was verified on M17 plates (results not showed). No influence of the bacteria was observed on the Ct values of phages. This indicated the ability of the method to measure exactly a given amount of phages within the sample without interference from nontarget contaminants on matrix. The composition of the whey did not seem to affect the amplification of bacteriophage by real-time PCR as observed for the milk (results not shown).

The reproducibility of the method was tested in goat's raw milk and other whey samples of cow's milk. Similar results were obtained (data not shown).

The standard curves established were used to detect and quantify the bacteriophages of the three bacteriophage groups of *Lactococcus* c2, 936, and P335 in various goat's raw milk and whey.

### 3.3. Detection of the Lactococcal Bacteriophage in Whey and in Goat's Raw Milk

**Figure 7:**
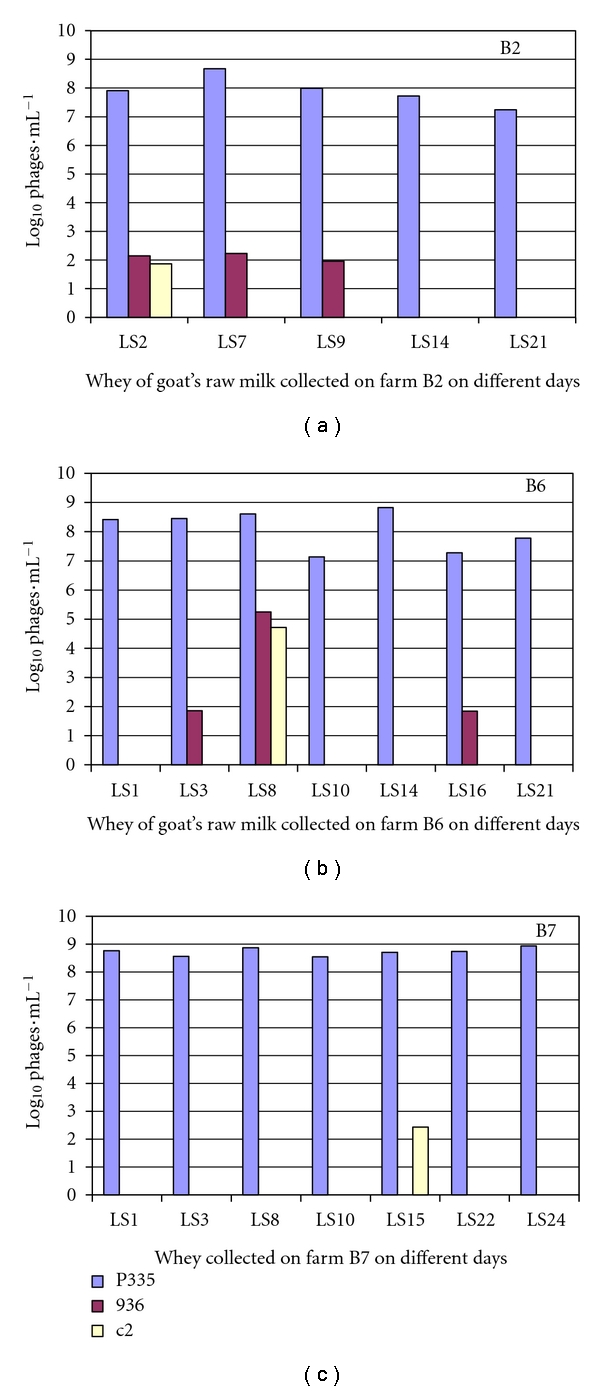
Detection and quantification of three main groups of bacteriophages of *Lactococcus *in whey of goat's milk collected on different days (LS) of three farms in Rhône-Alpes: B2, B6 and B7.

**Figure 8:**
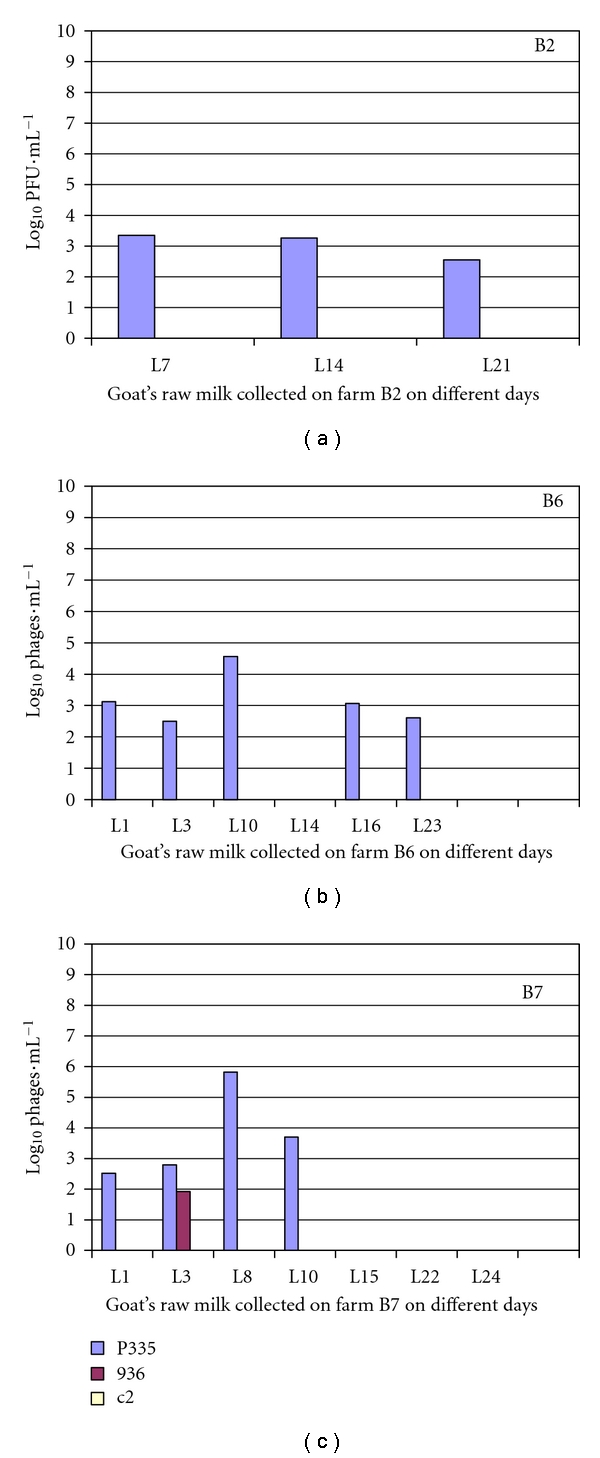
Detection and quantification of three main groups of bacteriophages of *Lactococcus *in goat's raw milk collected on different days (L) on three farms in Rhône-Alpes: B2, B6 and B7.

The real-time PCR system developed above was applied to detect the bacteriophages in whey and raw milk provided by three farms in the Rhône-Alpes region. In each farm, milk samples (coded by L) and whey samples (coded by LS) were collected on different days. DNA was extracted from 1 mL of sample and then subjected to amplification through real-time PCR. In all cases, we obtained a melting curve of 81.5°C, corresponding to bacteriophages. Nonspecific amplifications were present, that is, bacterial DNA. The concentration of each bacteriophage group was determined by the Ct values corresponding to the standard curves (Figures 7 and 8). All samples of whey and milk, except milk L14 from farm B6 and milk L15, L22, and L24 from farm B7, were contaminated by P335 bacteriophages. Concentrations found varied from 10^7^ to 10^9^ phages/mL in the whey of the farms B2 and B6 and from 10^7^ to 10^8^ phages/mL of the farm B7. The pH of the whey was about 4.5. The concentration of P335 in the milk was lower than in whey. Only 3 · 10^3^ phages/mL, 6 · 10^4^ phages/mL, and 9 · 10^5^ phages/mL were found in the milk of the farm B2, B6 and B7, respectively. 

Six whey samples and 1 milk sample from a total of 19 samples were contaminated by a weak quantity of 936 phages. Only 10^2^ phages/mL of the group 936 were detected in whey LS2, LS7, and LS9 of the farm B2 and LS3, and LS16 of the farm B6 as well as the milk L3 of the farm B7. The whey LS8 of the farm B6 was more contaminated with the 936 phage (10^5^ phages/mL). The phages of the group c2 were detected in three samples: the whey LS2 of the farm B2 and LS15 of the farm B7 at 10^2^ phages/mL and in the whey LS8 of the farm B8 at 8 · 10^4^ phages/mL. 

The phage of P335 group was predominantly found in samples with a greater concentration in the whey. The bacteriophages in whey sample LS2, LS7 of the farm B2, and the LS8 of the farm B6 were isolated. The c2 and 936 were isolated and verified by conventional PCR. The c2 and 936 were lytic phages with the concentration detected by double layer similar to the concentration detected by real-time PCR. P335 was not detected by the spot method among the host bacteria used. (Data not showed).

## 4. Discussion

The detection of bacteriophages in milk and whey is necessary to prevent infection of lactic acid bacteria during fermentation. The bacteriophages of *Lactococcus* can be detected by PCR multiplex PCR and [6, 13, 14]. According to these authors, the bacteriophages were first isolated on agar plate by double layer methods. The phages were then incubated in M17 and concentrated for the PCR. The time-frame of these analyses is from 24 h to 48 h. However, milk industry cannot immobilize the milk during the whole period analysis. Real-time PCR is known as a rapid technique to detect specifically the microorganisms with a better sensitivity than conventional PCR. This method was developed for the detection of c2 and 936 phages group from airborne in cheese plants [15] but not yet for bacteriophages of *Lactococcus *in milk and whey. In this study, we optimized the real-time PCR method which enabled to detect directly from whey or milk three groups of bacteriophages predominantly found in the milk industry c2, 936, and P335. 

The selection of the primers is critical for PCR amplification. del Rio et al. [6] have successively carried out the design of the primers to detect the main groups of phage c2, 936, and P335 by multiplex PCR. In this study, we used these primers to validate the program of PCR real-time with SYBR Green in optimizing the hybridization temperatures and the concentration of primers. The extraction protocol was then developed to detect directly the bacteriophages from raw milk and whey. The correlation coefficient (*R* ^2^), the slopes of curves and the PCR amplification efficiencies obtained for the three groups of bacteriophages were similar to those of other study on the detection of microorganisms by real-time PCR [11, 12, 16]. A standard curve with a slope of −3.33 corresponded to a reaction with an amplification efficiency value of 100%. The amplification efficiencies in detection by the real-time PCR are acceptable from 84% to 110% (according to Biorad).

Similar standard curves obtained from phage DNA extracted from artificially contaminated whey with bacteriophages and from pure cultures allowed estimating the performance of the extraction. In fact, the milk and whey are complex matrix containing inhibitory substances such as calcium, proteins, and fat causing a failure in the PCR amplification. Therefore, quality and purity of DNA extraction are primary requirements for a PCR-based detection assay. The extraction method is a determining factor for a successful and valid PCR analysis [17]. In this study, no interference of matrix and DNA contaminants was observed. According to del Rio et al. [6], the sample volume in PCR reaction mixture is critical. The increasing of DNA sample volume may decrease the detection sensitivity because of the increase of the inhibitor concentration. We tested increasing the DNA sample volumes (2 *μ*L or 5 *μ*L) instead of 1 *μ*L in the same final volume reaction (25 *μ*L). No inhibition of PCR reaction was observed. The extraction method in this study allows the increase of detection limit by increasing the sample volume. 

We analyzed the Ct value from milk and whey sample containing minimum 10^2^ PFU/mL in completing a final volume reaction of 25 *μ*L containing 1 *μ*L of DNA sample. This DNA sample was done in 100 *μ*L buffer after an extraction of 1 mL of milk or whey. In theory, the detection limit is one genome copy per reaction. Verreault et al. [15] detected the airborne c2 and 936 bacteriophages species in a cheese factory by real-time PCR. These authors have detected from 1 to 50 genome copies per reaction (25 *μ*L) for the 936 and from 5 to 50 genome copies for c2. It means that the detection limit varied from 2 · 10^2^ PFU/mL to 10^4^ PFU/mL for the 936 and from 10^3^ PFU/mL to 10^4^ PFU/mL for the c2. del Rio et al. [6] had similar detection limit. These authors detected the *Lactococcus* bacteriophages in complex matrix such as the milk by multiplex PCR. The detection limit was 10^4^ phages/mL for P335 and c2 and 10^3^ phages/mL for 936. Martín et al. [11] developed real-time PCR to detect the *Lactobacillus delbrueckii* bacteriophages in milk with the detection limit of 10 copies per reaction which is the equivalent to 10^5^ PFU/mL in milk. The extraction method and real-time PCR program of our study offer better detection limit than previous studies.

Among the sample collected from different farms in the Rhône-Alpes region at different days, P335 was the group of phages predominantly found. The concentration of this phage group was more important in whey than in raw milk. Studies on the bacteriophage biodiversity in the dairy industry showed that c2 and 936 groups represented the most frequently detected in cow's milk [10, 18]. Several factors could influence this difference. The samples of milk and whey come from a distinct geographical zone. The climate with the variations of temperatures can influence the development of the phages. The utilization of whey as natural starter may keep the phages contamination. On the other hand, working and sanitary conditions could result in the appearance of new phages. In the case of P335, the genome is organized in a mosaic form. The data from sequencing revealed that phages P335 shared only 10–33% of homology [19]. This genetic diversity is the probable consequence of recombinations inside the cell genome bacteria with that of the prophage [20]. The genome plasticity is likely to enable P335 to adapt to a new environment, including phage resistance mechanisms of [5]. 

The pH of whey was about 4.5 showing that the acidification was not perturbed in presence of P335 at high concentration. This result may be explained by two hypotheses. May be P335 is prophage or the milk was acidified by the acid lactic bacteria which were not infected by P335. It suggested that the use of a starter mixture of several lactic bacteria could mask the effect of bacteriophage on the acidification. In this case, despite the fact that the pH is not perturbed, one or several bacteria populations could be disappeared during the ripening of cheese.

## 5. Conclusion

To summarize, real-time PCR using the nonspecific fluorescent dye SYBR Green provides a sensitive, specific, reproducible, and rapid method for the direct detection of *Lactococcus* bacteriophages in whey and milk. One of the important steps in our study is the extraction of the DNA bacteriophage from complex matrices such as whey and milk. The extraction allowed the amplification of DNA bacteriophage in eliminating the inhibitor agents of PCR. The time-frame of the detection by realtime PCR in this study was of about 2 h. 

The next step will be to study the characteristics of bacteriophages found in goat's raw milk as their infection capacity and DNA profile. It would also be interesting to study the present of other bacteriophages in whey and in goat's raw milk.

## Figures and Tables

**Table 1:** Bacteriophages and host strains used in this study.

Bacteriophages	Source	Host strain	Source
c2 phage group			
c2	U.Laval	MG1363	INRA Dijon
p2	U.Laval	MG1363	INRA Dijon
bIL67	INRA Jouy en Josas	IL1403	INRA Jouy en Josas
P335 phage group			
P335	U.Laval	HER 1228	U.Laval
P270	U.Laval	HER 1228	U.Laval
sk1	INRA Jouy en Josas	MG1363	INRA Dijon
936 phage group			
936	U.Laval	HER 1203	U.Laval
P008	U.Laval	HER 1228	U.Laval
bIL170	INRA Jouy en Josas	IL1403	INRA Jouy en Josas

**Table 2:** Primers selected according to del Rio et al. [6].

Phage group	Primer	Sequence
c2	c2A	5′CAATCGAAGCAGGTGTAAAAGTTCGAGAAC 3′
	c2B	5′GCTTTATCCATTTGTAGGTATGCTTCTGCC 3′
P335	P335A	5′GAAGCTAGGCGAATCAGTAAACTTGCTAG 3′
	P335B	5′CGGCTATCTCGTCAATTGTTCCGGTTGC 3′
936	936A	5′ATCAGTTGGCTCAATGGAAGACCAAGCGG 3′
	936B	5′GTTGCTTCTGCTGTTGGTGTCAAATGAGGA 3′

**Table 3:** Results of real-time PCR performed at different hybridization temperatures.

Temperature	Efficacy (*E*) in %	Correlation coefficient (*R* ^2^)	Slope (*s*)
55	82,1	0,985	−3,730
58	85,7	0,991	−3,470
62	94	0,997	−3,475

**Table 4:** Results of real-time PCR tested with different bacteriophages.

Bacteriophages	Titration	qPCR result		
		c2 primers	936 primers	P335 primers
c2 phage group				
c2	1,5 · 10^5^ PFU/mL	10^5^ PFU/mL	No detection	No detection
p2	5,6 · 10^6^ PFU/mL	6 · 10^6^ PFU/mL	No detection	No detection
bIL67	2 · 10^5^ PFU/mL	1,58 · 10^5^ PFU/mL	No detection	No detection

P335 phage group				
P335	3,4 · 10^5^ PFU/mL	No detection	No detection	3 · 10^5^ PFU/mL
P270	4,4 · 10^6^ PFU/mL	No detection	No detection	4,5 · 10^6^ PFU/mL
sk1	6,7 · 10^5^ PFU/mL	No detection	No detection	7 · 10^5^ PFU/mL

936 phage group				
936	2,5 · 10^6^ PFU/mL	No detection	3,12 · 10^6^ PFU/mL	No detection
P008	5,2 · 10^5^ PFU/mL	No detection	5,34 · 10^5^ PFU/mL	No detection
bIL170	3,7 · 10^5^ PFU/mL	No detection	3,52 · 10^5^ PFU/mL	No detection
